# Suggestion in the Operation of Circumcision

**Published:** 1896-02

**Authors:** T. Richie Stone

**Affiliations:** Washington, D. C.


### SUGGESTION IN THE OPERATION OF CIRCUMCISION.

BY T. RICHIE STONE, M. D., WASHINGTON, D. C.

In the light of the modern advancement of surgery, with all its antiseptic precautions so necessary for success, many are apt to view with contempt this simple though essential operation, so pardon may be granted in using the old adage of "anything worth doing, is worth doing well."

The operation is one which I have performed many times and is essentially the same presented before the Clinico-Pathological Society of this city some years ago, but now revised with the modifications which experience has given.

In its performance a little more time may be expended, but wisdom gained by practice has demonstrated that the result amply compensates for the trouble. In making the markings for cutting, any amount of the redundant prepuce may be included, but, of course, enough tissue must be removed to overcome the stenosis present.

*Operation.*—The penis after being made practically aseptic and dried with sterilized cloths, is marked, in the flaccid state, externally from a point on the dorsum from about the middle of the coronal ridge round and downward to the frenum. The frenum is located through the medium of feeling the band-like insertion, or when the prepuce is pulled forward a needle can be stuck through from the internal to the external side (at the internal insertion) and thus form a guide, or, after the first dorsal incision is made, any correction in the external marking can be perfected. This is the line of incision.

Your assistant having applied to each side of the prepuce (dorsum) locked forceps, now pulls the prepuce as far forward as possible, at the same time dilating the orifice. The first cut is made while on the stretch in a line down the middle of the dorsum of the prepuce to the point marked on the corona, the two flaps are pulled aside and any existing adhesions broken down.

The two flaps made by the middle incision of the prepuce are firmly held apart by locked forceps such as hæmastatic. This is the most important step in the operation, and if not strictly observed, the object is lost. The reason for the locked forceps on the flaps is, that the under or mucous layer may be stretched upon the upper or dermoid layer, so that there will be no rolling or movement of the two layers, but will be firmly fixed on each other.

The next step is to put in the sutures while the flaps are on the stretch, beginning at the median line and following the external marking, and carried downwards and around to the frenum. They should be placed close together.

Here I must say that too few sutures are many times the cause of the malformations which should be carefully guarded against. The sutures (which are cut long) being in place, and caught at each end so as to cause no tangled obstruction, the half flap of redundant prepuce is cut around, following the marked external line. The other side is treated in the same manner, bleeding vessels ligated, sutures tied, dry dressings put on, and your operation is finished.

In doing this operation, as much of the prepuce may be removed as desired, and I quote from a paper by Dr. H. G. Howse in a "Note on the Operation of Circumcision in the Adult," in Guy's Hospital Reports, London, 1873, third series, vol. xviii: "The frenum may be looked upon as a mere fœtal remnant, having no very definite function. On the other hand, its presence is sometimes decidedly prejudicial." Economy in sparing the tissues of cutting the frenum does not play such an important part, for usually the amount left in many of the operations for phimosis leaves the patient with a ridge or stump of amputated prepuce which neither enhances the beauty nor usefulness of the organ; hence would suggest leaving the glans uncovered.

Again, many operators usually include bleeding points in their sutures, but if all bleeding points are carefully attended to before the sutures are finally closed, the operator would not be troubled by the midnight bell with its hurried summons.

All that is claimed is:

First. Quickness—the one cut (of the divided prepuce) being all that is required; obviating the necessity of trimming the under or mucous layer after the clamp is removed.

Second. The approximation or coaptation of the two edges are better acquired and there being no necessity for lost time in putting in sutures in the two surfaces far apart.

Third. The needles are put through with more ease, as both surfaces are together on the stretch.

Fourth. There being no cause to fear secondary hæmorrhage as all bleeding vessels are tied and the sutures being evenly and correctly put in, the bleeding, which is parenchymatous, is controlled.—*Maryland Medical Journal.*

